# Measuring growth: descriptive or prescriptive?

**DOI:** 10.1016/S2589-7500(19)30198-0

**Published:** 2019-11-07

**Authors:** James A Berkley

**Affiliations:** KEMRI/Wellcome Trust Research Programme, Clinical Research, CGMRC, Kilifi 80108, Kenya

In this issue of *The Lancet Digital Health*, Barbara Heude and colleagues^1^ describe the creation of paediatric growth charts by taking the novel approach of leveraging a massive and up-to-date dataset recorded in the course of routine clinical practice by French primary care physicians.

Growth is a reflection of health and the balance between the supply and demand for macronutrients and micronutrients. An observed value for body size (ie, anthropometry) or rate of growth is at its most useful when it is compared with reference data indicating how individuals normally grow with age or how different measurements relate to each other (eg, body-mass index [BMI] in kg/m^2^). However, how individuals normally grow is not easy to define. It can mean either how individuals usually grow (ie, descriptive references) or how individuals should grow (ie, prescriptive standards).

Prescriptive standards aim to be derived from measurements from optimally healthy individuals without nutritional limitations, disease, or exposures known to adversely influence growth. The leading example is the WHO Multicentre Growth Reference Study (MGRS). In this logistically challenging study, well controlled and standardised longitudinal and cross-sectional assessment was undertaken of infants and children from birth to 71 months of age.^2^ Participants were identified in affluent neighbourhoods in the USA, Norway, Brazil, Ghana, and India. Inclusion criteria included that mothers were or had followed WHO breastfeeding and complementary feeding recommendations, and there were no known health or environmental factors that could constrain growth such as maternal smoking. For the longitudinal assessment, 83% of screened participants were excluded. Remarkably, within these different locations and ethnic groups, growth was virtually identical.^3^

More recently, a similar approach was taken by the INTERGROWTH-21st study to create standards for fetal growth and growth of preterm infants using carefully standardised, prospectively collected data from advantaged populations in the UK, USA, Italy, India, China, Pakistan, Brazil, Kenya, and Oman. Again, growth was observed to be the same across all sites.^4^

These two studies strongly suggest that without environmental, nutritional, and socioeconomic restrictions (including intergenerational factors), the vast majority of human populations grow in the same way. Hence, the standards created are regarded as prescriptive for how children should grow and can be used as a target for attainment worldwide. No similar study has been done among school-aged children and adolescents—such a study would be complex and expensive.

Concerns regarding prescriptive growth standards include uncertainty as to whether they apply to all populations. We know that substantial differences in adult height and weight exist, including between high-income countries. There have been varying gains in height in the past 100 years,^5^ with gains in many countries continuing after the WHO MGRS data were collected in 1997−2003.

Descriptive studies aim to accurately represent the population of a country or region. They also generally apply criteria to exclude individuals with chronic disease or genetic variations affecting body size. However, where a study population is not optimally healthy or is partly wasted, overweight, or stunted, development of descriptive reference would be undesirable as they would provide unhealthy attainment targets. National growth charts based on descriptive studies are commonly not updated frequently, so might also not reflect current populations.

Barbara Heude and colleagues^1^ have developed a sophisticated automatic cleaning algorithm to model descriptive growth curves, excluding children with low birthweight; children with an excessive number of measurements, thus suggesting chronic illness; and to deal with outlying measures and detect repeated measures that were dissimilar.^6,7^ The authors have shared the algorithm and modelling code online. Advantages of this approach are low cost and timeliness. A potential risk of this approach is bias if the individuals using clinical services, or those being measured when they do attend, are unlike the general population. The essential step of external validation was undertaken, comparing the new growth charts with the WHO MGRS growth charts and existing French national growth charts. The modelled curves were much closer to the WHO curves than the existing French growth charts. When compared with national school survey data, there were significant discrepancies for weight, with the school survey data being higher than the predicted weight for both sexes during adolescence, probably reflecting prevalent overweight and obesity. Modelled height was generally closer to the survey data than the French national charts and the WHO growth charts, and the data suggest that updating national height references could be considered. However, the obesity epidemic was evident in these models, suggesting they should not be used to update weight references. A potential problem with updating height references only is that is it might also affect weight-for-height or BMI references.

The approach of mining existing large-scale health data in this way has a broad application. The ideal is to be linked to a subsequent health outcome where electronic records exist, such as anthropometry and, later, non-communicable diseases or survival. This might require very long-term data but overcomes the debate of what is considered normal by defining risks rather than normality. Whether with or without a health outcome, data collected for another purpose must always be handled with care, since, as nicely illustrated by Heude and colleagues, recognising and limiting bias is crucial.
